# Proof of concept study: Testing human volatile organic compounds as tools for age classification of films

**DOI:** 10.1371/journal.pone.0203044

**Published:** 2018-10-11

**Authors:** C. Stönner, A. Edtbauer, B. Derstroff, E. Bourtsoukidis, T. Klüpfel, J. Wicker, J. Williams

**Affiliations:** 1 Max Planck Institute for Chemistry, Mainz, Germany; 2 Department of Computer Science, The University of Auckland, Auckland, New Zealand; University of Alabama at Birmingham, UNITED STATES

## Abstract

Humans emit numerous volatile organic compounds (VOCs) through breath and skin. The nature and rate of these emissions are affected by various factors including emotional state. Previous measurements of VOCs and CO_2_ in a cinema have shown that certain chemicals are reproducibly emitted by audiences reacting to events in a particular film. Using data from films with various age classifications, we have studied the relationship between the emission of multiple VOCs and CO_2_ and the age classifier (0, 6, 12, and 16) with a view to developing a new chemically based and objective film classification method. We apply a random forest model built with time independent features extracted from the time series of every measured compound, and test predictive capability on subsets of all data. It was found that most compounds were not able to predict all age classifiers reliably, likely reflecting the fact that current classification is based on perceived sensibilities to many factors (e.g. incidences of violence, sex, antisocial behaviour, drug use, and bad language) rather than the visceral biological responses expressed in the data. However, promising results were found for isoprene which reliably predicted 0, 6 and 12 age classifiers for a variety of film genres and audience age groups. Therefore, isoprene emission per person might in future be a valuable aid to national classification boards, or even offer an alternative, objective, metric for rating films based on the reactions of large groups of people.

## Introduction

With box office revenues worldwide estimated to be around 40 billion US dollars,[1] the global film industry is an important element of many national economies. Once a film is recorded and edited it is must be classified prior to distribution to the cinemas. Movie classification serves to protect children from unsuitable media content and to inform consumers, particularly parents, of the film´s subject material. This classification is made at the national level by an independent regulator according to guidelines based on the legal framework of the individual country. The regulator assigns a rating to the film that reflects the public´s sensibility to the film´s content, ranging from unrestricted (suitable to all) up to adults only (typically 18 years old). The division of the classification system into age groups varies greatly from country to country. For example, Germany uses 0, 6, 12, 16, 18,[2] while the United States has G (general audiences), PG (parental guidance suggested), PG-13 (parents strongly cautioned), R (restricted) and NC-17 (no one 17 and under admitted).[3] India the world´s most prolific film maker, uses U (0 to 11), UA (to 17) and A for adult.[4] The classification process is complicated by the numerous influencing factors that must be considered together before the age classifier can be assigned, such as the degree of violence, sex, antisocial behaviour and bad language.[5] Furthermore, public opinion on certain aspects of the classification guidelines may change with time requiring the regulator to revise their guidelines regularly. Ultimately, the classifying authority expresses a subjective assessment on behalf of the public in the form of an age limit.[6] On some occasions this can be a contentious decision as a film maker seeking a larger market for their film may consider their work suitable for a broader audience than the classifying agency.

Clearly, it would be helpful to classification authorities if objective data based methods could be used to support the decision. Recently it was shown that cinema audiences emit chemical signals into the surrounding air in response to specific scenes in a film. Moreover, the sequence of signals over time was reproducible over multiple screenings of the same film.[7,8] The effect can be most easily understood in terms of carbon dioxide (CO_2_), which makes up circa 4% of exhaled human breath. Cinemas are ventilated continuously with outside air containing circa 0.0004% CO_2_ so that when an audience is present the CO_2_ level rises smoothly until an equilibrium is reached. However, when audience pulse and breathing rates increase momentarily in unison, in response to a particularly exciting scene, a peak in the CO_2_ is generated which can be detected in air vented from the cinema. Current air measurement technology allows, in addition to CO_2_, several hundred volatile organic compounds to be measured at high frequency (every 30 seconds). In the aforementioned study, it was found that certain chemicals corresponded to specific scene types, with suspense and comedy scenes being best characterized. The chemical response measured in the “crowd breath” represents the reaction of a large group of people to the scenes shown.[7,8] This information could be potentially very useful in film classification as the chemical information is a direct, non-invasive measure of how a large group of people react to particular scenes and to the film as a whole. It is easy to imagine that the variability in the CO_2_ trace, the number of peaks in the individual VOCs or the absolute amounts of the chemicals emitted per person are all possible indicators of the group response.

Recently, computerized systems evolved to support the decision making of the age rating of a film by the committee. Most of these methods utilize the language (use of bad words) and the image properties (colour variance, shot length) to classify the films [9,10] but do not take into account the human reaction to the film.

In this study we systematically examine the feasibility of using CO_2_ and over 60 VOCs measured in air ventilating from a cinema to classify films. The assessment is based on 135 screenings of 11 different films collected over 8 weeks from two separate cinemas involving more than 13000 people. Our approach involves a random forest model built with time independent features extracted from the time series of every measured compound for every film. These features include for example peak height, peak width and the number of peaks in a film normalized to its length.[11,12] Finally, a permutation test was performed to test the resulting performance measures (area under ROC curve) of the original model versus the ones calculated from randomized class labels.[13]

## Materials and methods

### Cinema measurement

We are very grateful to the Cinestar company for permission to use their facilities. No specific permission was required. Individual audience members were neither harmed or identifiable in the gas mixture and therefore the measurements were not subject to ethical approval.

The measurements were conducted in the multiplex cinema Cinestar in Mainz, Germany (located at 49° 59' 37.511" N 8° 16' 45.548" E) in two different screening rooms for approximately four weeks during the winter 2013/2014 and winter 2015/2016. Over the 8 weeks of measurement, 11 different films were shown multiple times resulting in a total of 135 separate screenings. Table 1 summarizes the films measured, categorized according to the German film classification system age recommendations “FSK” (“Freiwillige Selbstkontrolle der Filmwirtschaft” meaning voluntary self-regulation) along with the number of screenings. The average number of people present at each screening is given in the supplementary S1 Table. It can be seen that each age recommendation class was attended by approximately the same amount of people.

**Table 1:** Summary of the measured films partitioned into the four different age recommendation classes.

	FSK 0		FSK 6		FSK 12		FSK16	
	Help I’ve shrunk my teacher	18	Buddy	10	The Starving Games	2	The Counselor	1
	I’m off then	33	Walking with Dinosaurs 3D	12	Hunger Games: Catching Fire	8	Machete Kills	1
			The Secret Life of Walter Mitty	13	Star Wars: The Force Awakens	34	Paranormal Activity: Ghost Dimension	3
**total**		51		35		44		5

For this study, the German motion picture rating system was used dividing the films in 5 categories. Unrestricted films are classified as “FSK 0”, films released to 6-years-old and over as “FSK 6”, films released to 12-years-old and over “FSK 12”, films released to 16-years-old and over “FSK 16” and films allowed only to adults “FSK 18”. During the period of measurement, no film with the age rating “FSK 18” was screened. Since children under 12 have a discounted ticket price, the proportion of viewers at a particular film under 12 could be taken from the ticket sales.

The two different screening rooms were approximately the same size with a seating capacity of 237 and 227 viewers respectively. The size of the screening rooms was 6500 m^3^ and the rooms were continuously flushed with 1300 m^3^/h fresh outside air. No internal influx of consumed air from the cinema was mixed with the fresh outside air. The entire exhaust air of the screening room was drawn through a 75x75 cm stainless steel ventilation shaft. The air from the exhaust shaft was measured in a separate technical room with a PTR-TOF-MS and a CO_2_-Analyzer.

### Proton transfer reaction time-of-flight mass spectrometer

The exhaust air of the cinema was measured with a PTR-TOF-MS 8000 (Ionicon Analytik GmbH, Innsbruck, Austria). The ionization of each analyte occurs via hydroxonium ions (H_3_O^+^) resulting in protonated positively charged ions. This transfer reaction proceeds only to molecules with a higher proton affinity than water (691 kJ/mol). Thus the system is blind to the main air components like nitrogen, oxygen and argon. The low energy involved in the protonation reaction results in small fragmentation of the analyte facilitating identification.

A detailed description of the set up and the calibration can be found elsewhere. [14]

### Data analysis

In total, 20% of the film screenings measured had to be discarded due to problems associated ventilation system checks around midnight (only in winter 2013/2014) and high VOC emissions from cleaning products in the morning masking some human emissions (only for pre-midday screenings).

The data analysis was divided up into a pre-processing step and a model building step. The latter includes the generation of instances and the partitioning into training and test sets. Finally, the resulting performance measures were compared to the results obtained from a permutation test.

#### Pre-processing

**Fig 1:**
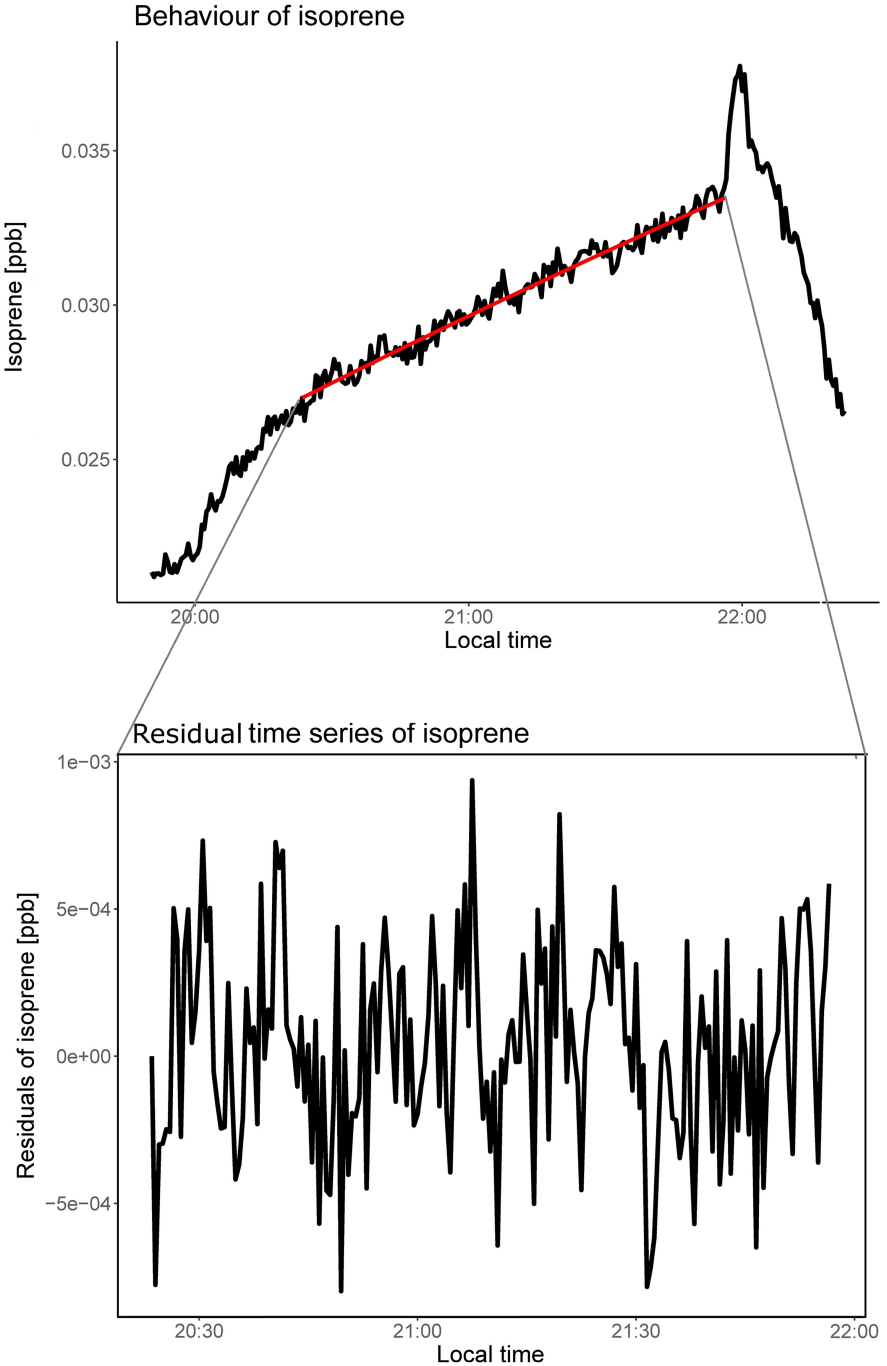
Time series of isoprene. The upperpanel shows the mixing ratio of isoprene (in ppb) during the film "I’m off then". This time series was already divided by the number of viewers. The black line shows the measured values and the red one the modelled mixing ratio assuming a constant emission rate of isoprene. The lower panel shows the residuals obtained by subtracting the measured times series from the modelled one.

The measured time series for the isoprene mixing ratio for one film (“I´m off then”, “FSK 0”) is shown as the black curve on the left side of Fig 1. As the audience enters the cinema, the mixing ratio of isoprene increases quickly at first and then steadily during the film before decreasing sharply at the end when the audience leaves the screening room. In the case of isoprene, the peak that can be seen at the end of each movie is caused by enhanced release of isoprene due to muscle contractions associated with standing up and walking out.[15,16] This peak was discarded for the analysis by removing the last 5 minutes of each film in the data pre-processing step.

Zooming in, several peaks and valleys can be seen which re-occur at the same time in every screening of the same film.[7] The maximum mixing ratio of the VOCs measured for each film, positively correlates with the number of viewers attending the screening room. Therefore, the time series of the individual films were normalized to the number of viewers which is known from the ticket sales. The temporal behaviour of isoprene with an initial sharp increase, followed by a smooth steady enhancement and final rapid decrease were similarly observed for many other breath-borne compounds such as CO_2_ and acetone. The increase in the mixing ratio (red curve in Fig 1) can be calculated using a box model assuming a constant emission rate during the film. Within the model the mixing ratio is dependent only on the inflowing and outflowing air and the emission rate of the VOCs from the audience. A detailed description of the model can be found in the supplement. The modelled behaviour of the mixing ratio assuming a fixed emission rate (red curve in Fig 1) was subtracted from the measured mixing ratio (black curve on the left side in Fig 1). The resulting trace without the increasing trend was termed as the “residual time series” and can be seen on the right hand side in Fig 1.

The corrected time series was used to extract distinctive features comprising standard deviation, skewness and kurtosis of the time series as well as several features describing the occurrence of peaks in the time series. Additionally, the mean of the positive and negative values was included into the feature set to obtain an overall measure of change within the time series. The residual time series allows the comparison between the peak heights of different films. In one case all peaks were counted (single time step increase and decrease). In a second case, peaks were only counted exhibiting a sequence of a minimum of 3 consecutive increasing and decreasing steps. In the case of the peak height and peak width only the 5 highest and widest peaks with a minimum of 3 increasing and decreasing steps were taken and included in the feature set. In total 18 features were included in the feature set. The complete list of extracted features can be seen in Table 2. These features were extracted for each film and for each measured VOC. A separate model was built for each of the measured 66 VOCs. The statistics of the data set are shown in the supplementary S2 and S3 Tables.

**Table 2:** Summary of the extracted features.

Extracted features
Moments: Standard deviation, Kurtosis, Skewness
Sum of all positive values
Sum of all negative values
Amount of peaks (all peaks and peaks with a minimum of 3 increasing and 3 decreasing steps) normalized to the film length
Occurrence of the first peak
5 highest peaks normalized per person
5 widest peaks normalized per person
sum of the 5 highest peaks
Sum of the 5 widest peaks

#### Model building

Instances were created for each molecule in the same manner using the four age ratings “FSK 0”, FSK 6”, “FSK 12” and “FSK 16”. For the modelling process, the films were divided up into a training and a test set. For each age recommendation class, one film was chosen to be in the test set and the remaining films were put in the training set. In order to receive statistically meaningful results, the test set contained only films with 8 or more recorded screenings. An exception involves the age recommendation “FSK 16” because two films were measured only once (“The Counselor” and “Machete Kills”). These two films were always put into the same set (training or test set) and were evaluated together. Consequently, the other set includes “Paranormal Activity”. This set up results in 24 combinations of different training and test sets (two possible films in “FSK 0”, “FSK 12”and “FSK 16” and three possible films in “FSK 6”).

For each training set a random forest model was constructed.[17] The random forest classifier was run with the default values for its parameter specifically the number of trees to grow was set to 500 and the number of variables randomly sampled at each split was set to 6 (number of variables divided by 3). This model was used to predict the age rating of the corresponding and unseen test set. The classifier performance was evaluated using Receiver Operating Characteristic (ROC)[18,19] and Precision-Recall curves (PRC).[20] The ROC curve with its corresponding area under curve (AUC) value is frequently used in the machine learning community. However, this performance measure lacks interpretability when it comes to imbalanced data sets.[21] In our study the number of negative examples exceed the number of positive examples. For instance, a large number of false positives weakly enhances the false positive rate used in ROC. On the other hand, the precision value is affected by a larger amount because this value compares the false positives and the true positives. Between the ROC and PRC curves a one-to-one relationship exists, meaning that each point in one curve uniquely corresponds to one point in the other curve and vice versa.[22]

#### Permutation test

A permutation test was performed to check for spurious results. For this test a random age rating was assigned to each film in the training set and the initial class distribution was retained. The test set kept the original age ratings. From the resulting model, the area under curve from the ROC and the PRC were calculated. For each test set composition the training set labels where shuffled 50 times and the resulting 50 performance measures where compared to the corresponding original performance measures. Therefore, the cases in which the performance measure of the permutation test exceeds the one of the original test set composition were counted and divided by the total amount of permutations. The calculation of the p-value was according to Ojala et al.[13]

Generally, a Holm-Bonferroni correction should be applied to the p-values to counteract the problem of multiple comparisons as in our case summarizing the models from all measured VOCs. In this study we did not apply this correction since we only searched for indications pointing to VOCs which might be useful for further analysis. Therefore, we used the uncorrected p-values.

## Results

The resulting AUC values are summarized in Table 3. The complete results with the corresponding standard deviation of the AUC values and p-values are given in the supplement (supplement S4–S6 Tables).

**Table 3:** Summary of several VOCs with the corresponding area under ROC curve for the different age classes. AUC values of 0.70 and higher were highlighted in bold font.

*Mass*	*Compound*	*FSK 0*	*FSK 6*	*FSK 12*	*FSK 16*
***CO***_***2***_		0.55	0.53	**0.75**	0.15
***m31.0178***	Formaldehyde	0.55	**0.71**	0.48	0.39
***m33.0335***	Methanol	0.50	0.62	0.36	0.26
***m45.0335***	Acetaldehyde	0.45	0.50	0.51	0.14
***m59.0491***	Acetone	0.55	0.63	0.56	0.13
***m61.0284***	Acetic acid	0.54	0.54	0.55	0.32
***m63.0263***	Methyl mercaptane / Dimethylsulfide	0.55	**0.76**	0.40	0.44
***m65.0215***		**0.74**	**0.79**	0.40	0.17
***m65.0604***		0.36	0.64	0.58	0.16
***m67.0542***		0.40	0.65	0.47	0.52
***m69.0699***	Isoprene	**0.84**	**0.74**	**0.70**	0.25
***m83.0455***		0.38	0.40	**0.70**	0.60
***m95.0855***		0.53	0.54	**0.70**	0.55
***m137.1325***	Sum of Monoterpenes	0.60	0.58	0.64	0.66
***m235.2056***		0.51	0.55	**0.73**	0.48
***m355.0698***	Fragment of Decamethylcyclopentasiloxane	0.54	**0.73**	0.59	0.38

It can be seen that most of the VOCs show AUC values below or around 0.5 indicating a performance similar to a random classifier. The AUC value for CO_2_ shows the highest value in the age class “FSK 12”, whereas almost no significance is seen in the other categories. Isoprene shows AUC values above 0.7 for the age classes “FSK 0”, “FSK 6” and “FSK 12”. The AUC value for the age class “FSK 16” lies below 0.5. However, this class is hardly interpretable since we measured only 6 films for this class. This may pose a problem to the reliable prediction of this class. Therefore, in the following discussion the age recommendation class “FSK 16” was omitted.

In general, it can be seen that based on the AUC values several different compounds are able to distinguish between one or more age classes. Isoprene, which is one of the main VOCs on breath, seems to be a potentially useful compound for the differentiation of the age classes FSK 0, 6 and 12. Other compounds being able to predict only one age recommendation class are for example CO_2_, formaldehyde and decamethylcyclopentasiloxane. Additionally, a filter for low attendance films was imposed and films with less than 10 (in total 5 films were excluded) and 20 (in total 8 films were excluded) people were removed from the data set. The resulting AUC values show that this removal does not affect the prediction significantly.

**Fig 2:**
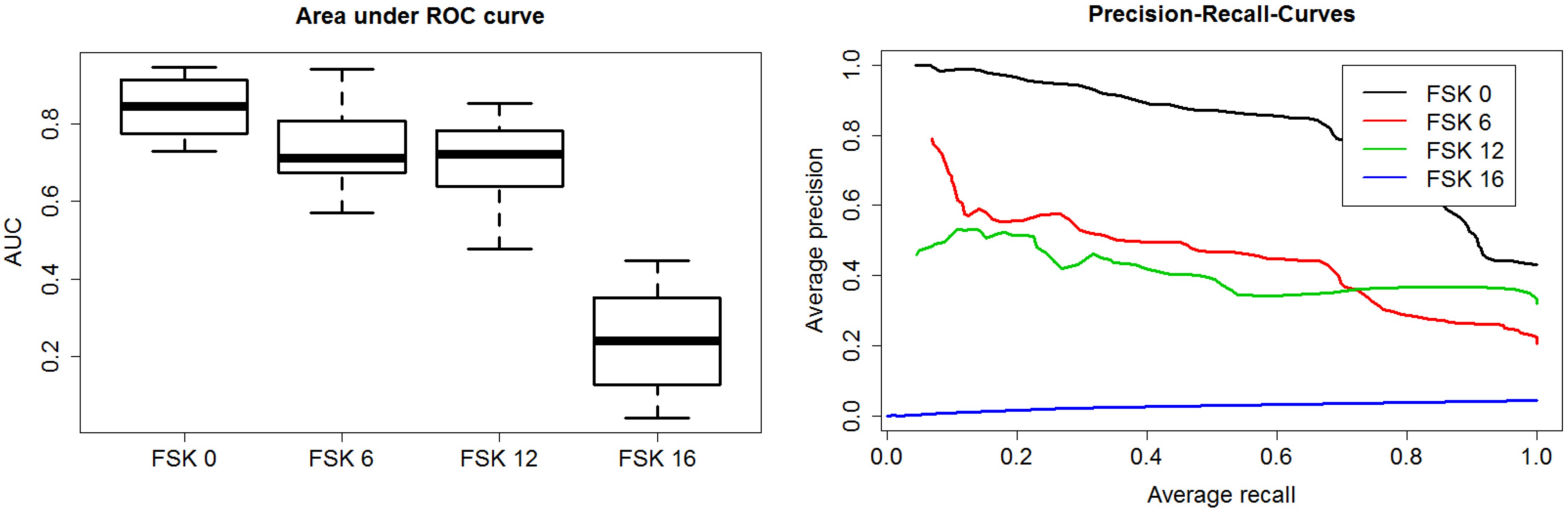
Performance measures for isoprene models. The box plots of the calculated area under ROC curve values(left) and average Precision-Recall curves for all age recommendations(right). The performance measures were derived from the isoprene models. The box plots on the upper left panel in Fig 2 the thick black line in the middle of the box indicates the median value for each group. The box comprises the interquartile range (IQR) of the data and the whiskers define 1.5 times the IQR or the minimum and maximum if no points exceed the 1.5 time IQR.

Fig 2 shows the average behaviour of the AUC values derived from the ROC curves and PRC for the age classes from 0 to 16 for isoprene. In general, 24 models were built for each measured VOC corresponding to the 24 different test and training set combinations. The plots in Fig 2 show the average value of the performance measures calculated from the 24 different test and training set combinations of isoprene. In case of the age class “FSK 0” all AUC values lie above 0.5 indicating a non-random classifier. For the age recommendations “FSK 6” and “FSK 12” some of the AUC values lie around the value of 0.5 (median AUC value ~0.70) indicating that some models trained and tested on particular sets cannot be predicted with a higher chance than a random classifier. The PRC shows the average curve over all models. The PRC curve shows a similar behaviour as the AUC values describing the age class “FSK 0” as the best predicted class followed by the age classes “FSK 6” and “FSK 12”. However, the age classes “FSK 6” and “FSK 12” exhibit higher average precision values of ~0.6 for lower recall values up to 0.2. The p-values from the permutation test for isoprene for the “FSK 0”, “FSK 6” and “FSK 12” are 0.01, 0.05 and 0.16 respectively. Consequently, for a significance level of α = 0.05 the null hypothesis for “FSK 6” and “FSK 12” cannot be rejected. Examining the variable importance for all 24 random forest models built for isoprene no specific feature was found to distinguish oneself from the others.

### Different genre labels

In this section we examine the differences in classifier performance between films of the same age class but with different genre label. The genre labels for the films were taken from the International Movie Database (IMDb).[23] For this purpose, the films of the age class “FSK 6” were selected due to their similar frequency in the number of screenings (10 films of “Buddy”, 12 films of “Walking with Dinosaurs 3D” and 13 films of “The Secret Life of Walter Mitty”). Here the film “Walking with Dinosaurs 3D” was labelled as “action” whereas “Buddy” and “The Secret Life of Walter Mitty” are “comedy” films.

**Fig 3:**
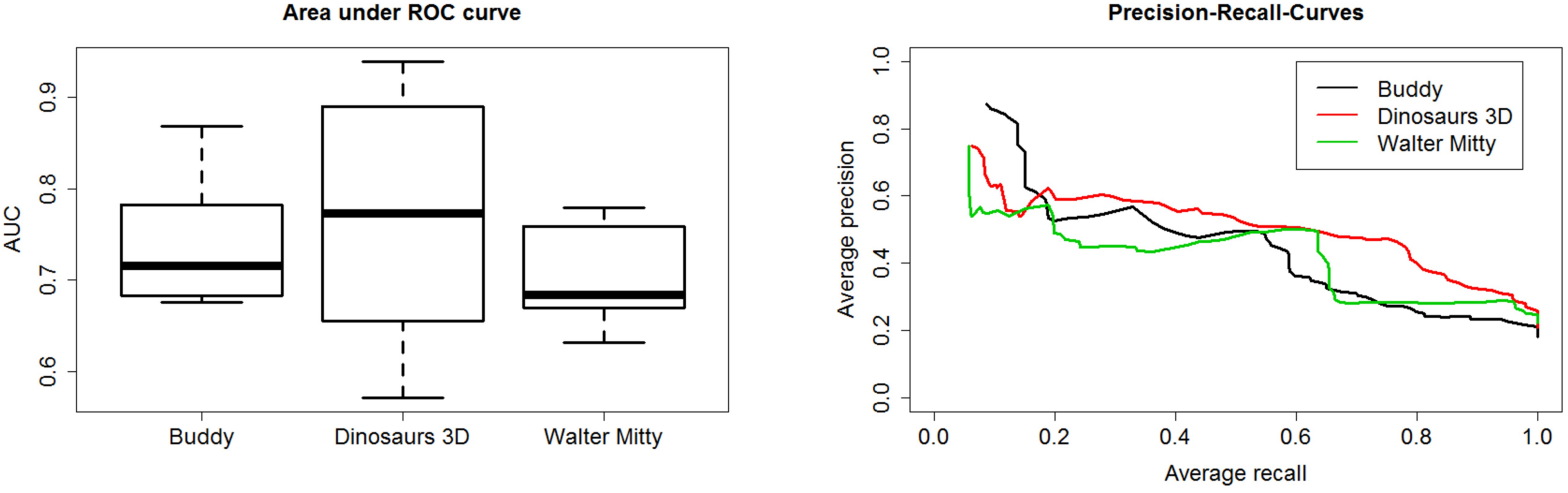
Performance measures for isoprene models involving only the “FSK 6” films. Area under ROC curve and Precision-Recall-curves divided up into the different films in the age recommendation "FSK 6". The film “Walking with Dinosaurs 3D” was labelled as “action” whereas “Buddy” and”The Secret Life of Walter Mitty” are comedy films.

Fig 3 shows the distribution of AUC values and PRC depending on the film in the test set. On the right side the results are shown for the age recommendation “FSK 0” and on the left side the results for “FSK 6”. Note that one film was chosen in the test set putting the other two films in the training set. It can be seen on the left side that the highest mean AUC value (~0.77 ± 0.13) is obtained placing the film “Walking with Dinosaurs 3D” in the test set. This test and training set combinations also shows the highest standard deviation. Fig 3 compares the mean PRC of the three films in the age class “FSK 6”. In general, all three PRC curves seem to behave similarly despite using films with different genre labels. Evaluating the permutation tests separately for the three different films results in p-values for the film “Buddy” to be 0.06, “The Secret Life of Walter Mitty” to be 0.04 and “Walking with Dinosaurs 3D” to be 0.11. In this case the p-values were calculated by comparing the original test set combinations in which the selected film appears with the corresponding randomized ones. These p-values are in accordance with the boxplot in Fig 3 showing the film “Walking with Dinosaurs 3D” with the highest standard variation. Thus, the chances are higher that the AUC values of the permutation test exceeds the values of the original one. It seems that some of the training and test set combinations do not predict the “Walking with Dinosaurs 3D” film well and that the genre label has an effect on the prediction results. However, the film “Walking with Dinosaurs 3D” could be predicted comparably well keeping in mind that there is no other action film in the training set if “Walking with Dinosaurs 3D” is in the test set.

### Different age of the audience

The ticket sales information provided the proportion of viewers younger than 12, to viewers 12 years or older. In the case of the age rating class “FSK 0” the films shown were aimed at quite varied audiences. The film “Help, I’ve shrunk my teacher” was classified as a “family” film by IMDB and the proportion of viewers younger than 12 was 64%. In contrast, the film “I’m off then” was attended only by viewers of 12 or older. The film “I’m off then” was more targeted to adults as it deals with a man on a pilgrimage in Spain. Additionally, it is known that the emission rate of CO_2_ is age dependent and rises until the age between 21 to <30 for male and female persons and then decreases again.[24] It was shown in a previous publication concerning the emission rates of VOCs that the emission rate for CO_2_ for the film “I’m off then” is higher than for the film “Help, I’ve shrunk my teacher”.[14] This gives an important hint that the average age of the audience is older for the film “I’m off then” than for the film “Help, I’ve shrunk my teacher”. Remarkably, this age rating could be predicted with the highest AUC of 0.84 despite the difference in the average age of the viewers.

**Fig 4:**
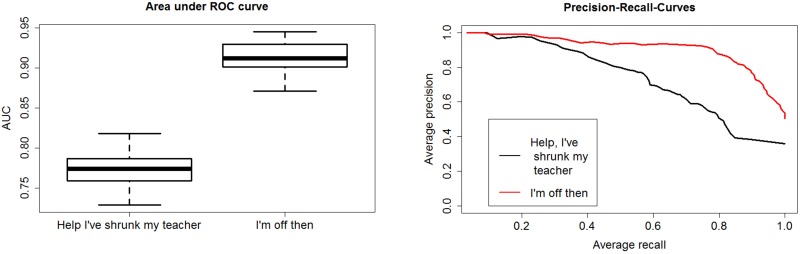
Performance measures for isoprene models involving only the “FKS 0” films. Area under ROC curve and Precision-Recall-curves divided up into the different films in the age recommendation "FSK 0". The film “Help I’ve shrunk my teacher” was attended by a large proportion of viewers younger than 12 (64%) whereas the film “I’m off then” was only seen by viewers of 12 or older.

The difference in the AUC value between those two films with the averaged PRC curve for each film can be seen in Fig 4 on the right side. Both PRC curves exhibit precision values higher than that of a random classifier. It can be seen that the classifiers perform worse, if the training set contains the film “I’m off then” and the test set “Help I’ve shrunk my teacher” than vice versa. Nevertheless, the age difference of the audience between those two films does not seem to worsen the classifier critically and the age class can be still predicted to a reasonable degree.

## Discussion

In this study we have assessed whether the age classification of a film can be predicted based on variations of airborne chemicals measured in a cinema. Previous publications[7,8] have reported correspondence between audio-visual stimuli and the emission of VOC from human beings. The aforementioned study reported that scenes labelled with “suspense” and “comedy” caused the audience to change their emissions of chemicals significantly. Intuitively then, we may think that these chemical changes may be related to the age classification given to the film. For example, films with horrific scenes will induce rapid pulse and breathing rates and hence higher and more variable levels of CO_2_. Indeed, CO_2_ values are effective in predicting FSK 12 films, probably because the films in this category were action films. However, our results show that most of the chemicals measured, including CO_2_, do not reliably predict all the age classifications of the films (0, 6, 12). One reason for this may be that current age recommendations for films are not solely linked to the intensity of induced fear, or the audience´s innate visceral responses to film content. Rather it is subjectively based on a synthesis of multiple aspects such as the degree and intensity of violence, sex, antisocial behaviour, drug taking and bad language. Provided that the audience´s reaction to these aspects of the film are in some way reflected within the large chemical dataset, an alternative and objective age classification may still be possible. It is interesting to reflect that a chemical based approach as advocated here, would be based on directly measured responses from large test audiences, whereas the current scheme is based on a subjective appraisal of the film by relatively few people entrusted to reflect the general public sentiment.

Of all species tested we found that isoprene performed best in predicting the different age classifications. The highest AUC value was obtained for “FSK 0” (AUC value of 0.84 ± 0.07). In this age recommendation class, the two films had a different proportion of younger viewers (in “Help I’ve shrunk my teacher” 64% of the audience was younger than 12 and in “I’m off then” only viewers of the age of 12 or older attended). It is known, that the children emit significantly less isoprene than adults.[14,25] Nevertheless, it was found that the age structure of the audience does not critically worsen the predictive power of the classifier and that the features displaying the structure of the films were able to distinguish this class from the rest. Lower precision values were reported for the age recommendation classes “FSK 6” and “FSK 12”. For these two classes the p-value of the permutation test lies between 0.05 for “FSK 6” and 0.16 for “FSK 12”. The age recommendation “FSK 6” included three films with similar frequency of two different genre labels (two comedy films and one action film). Again, this does not seem to influence the classifier’s performance. In the case of “FSK 12”, the average AUC values were 0.63 ± 0.10 if the film “Star Wars” is included in the test set (and the films “The Hunger Games” and The Starving Games” in the training set), and 0.77 ± 0.05 if the film “The Hunger Games” is included in the test set (the films “Star Wars” and The Starving Games” in the training set). The lower AUC (0.63) value and the higher corresponding p-value of the permutation test (0.25 versus the p-value of 0.08 including “The Hunger Games” in the test set) is likely due to the lower amount of training examples (12 screenings with “The Hunger Games” and the “Starving Games” in the training set).

**Fig 5:**
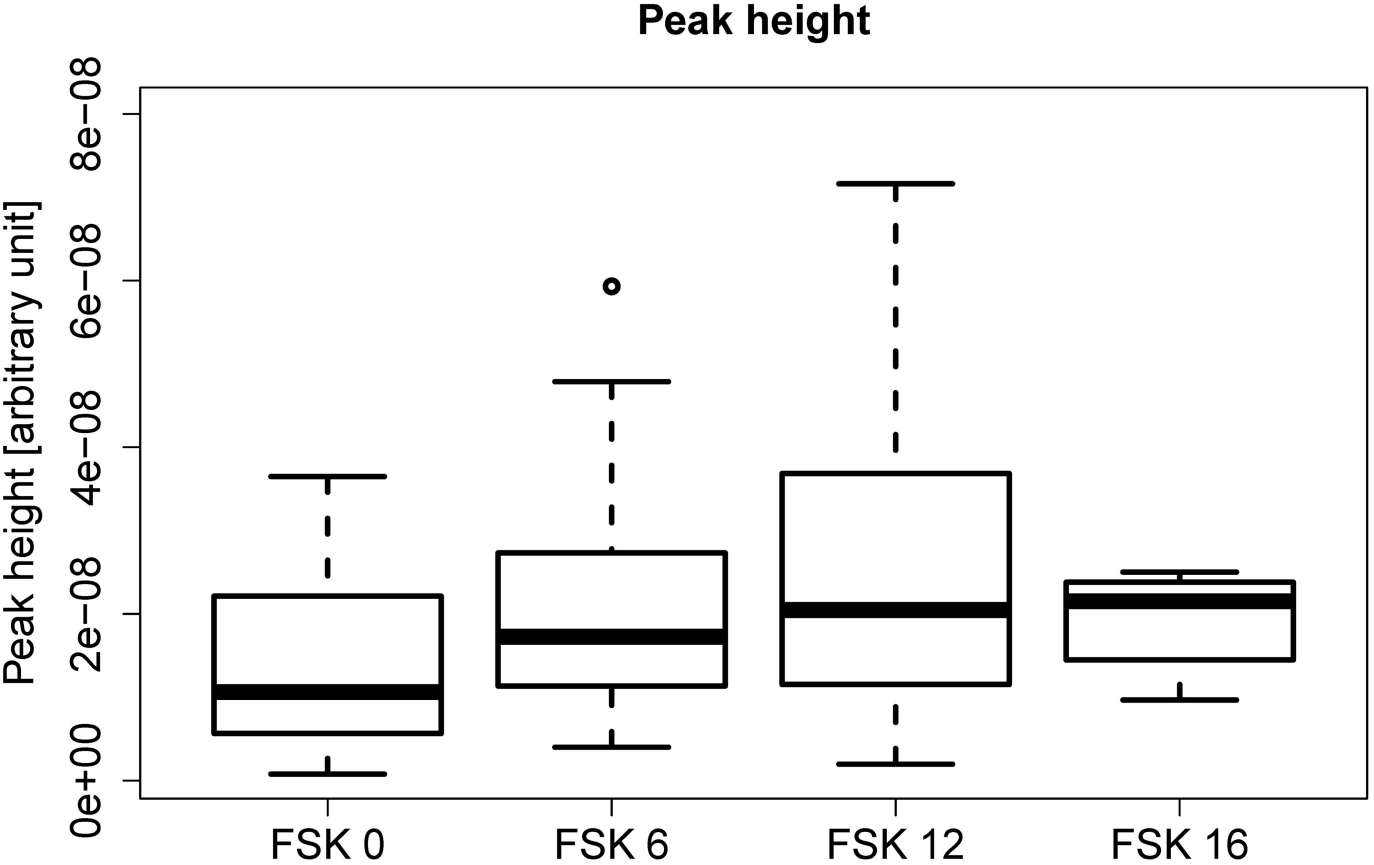
Boxplot with the height of the highest peak for the different age recommendations.

Isoprene is generated in the body during cholesterolgenesis[26] and stored in muscle tissue. Muscle movement causes stored isoprene to enter the bloodstream and then vent the body via the breath.[15,16] It is interesting that that this species, rather than CO_2_, can be used as a successful delineator for film classification, at least for FSK 0,6, and 12. Generally, it could be seen that isoprene was reproducibly emitted at higher rates at the same time point in the same film, even with different audiences. The height of peaks depicted in Fig 5 show smallest values for the age class “FSK 0”. This could be because of the lower concentration of isoprene in the breath of children for the film “Help I’ve shrunk my teacher” or because of fewer suspense scenes in both films. Suspense scenes generally lead to increased heart and breathing rates as well as involuntary movement, all of which enhance the isoprene emission rate of the audience. We may speculate that in future isoprene may be used to objectively classify films, using the dataset shown here as the basis or conceivably, measurements from a test audience could be used by the classification board to aid in decision making in borderline cases.

The presented findings imply that features within the isoprene trace correspond to the pattern and the intensity of induced emotions in the film. This structure can be distinguished from the other classes even when the audiences between the films in the same class consists of different age proportions (“FSK 0”) or the genre labels of the films are different (“FSK 6”). In general, the isoprene trace properties should reflect the subjective assessment of the rating agency (age classification). It would be interesting to see if isoprene acts as an indicator in other domains with multiple underlying stimuli like psychological stress. The perception of stress can also be caused by several environmental conditions and events.

In this study we used a random forest model built for each measured mass separately to predict the age recommendation of a film. Future work should involve combinations of different masses. As shown in Table 3 masses like m65.0215 or CO_2_ show higher AUC values for the specific age classes like “FSK 6” (AUC of 0.79 for m65.0215) and “FSK 12” (AUC of 0.75 for CO_2_) than isoprene. These masses might respond better to specific scenes or capture similar structures of films within the same age recommendation class. Thus, the combination of the features of these masses might help to reflect the structure of the film and to improve the classifier’s performance. This approach requires a larger number of measured films because as in our case the classifier adopted the features of the few films in the training set very well (resulting in a perfect classification for the training set) so that the extrapolation to new films in the test set resulted in comparably low performance values. The addition of new features from other masses exacerbates this problem. Therefore, a new validation set should be used to choose the best combinations of these masses and to test them on an unseen set of films. Therefore, future datasets should include a larger number of films.

## Conclusion

This study presents a framework to objectively gauge a film into an age classification system based on VOCs and CO_2_ in cinema air. The evaluation of these compounds resulted in no single human generated volatile compound being able to distinguish all four age classes (0, 6, 12, 16). Problems arise because of the small number of available films within each age class, especially for the age class “FSK 16”. Overall, the results of this first study are promising for isoprene. Perhaps in future metrics can be devised using combinations of isoprene and other VOCs to designate movie classification. This could be useful for the film industry which edits films to make them accessible for their desired target audience. It can be seen for the age recommendations “FSK 0” that the classification is based on the films in this class and not on the age structure (different target audience in “FSK 0”), so the classifying ability is not based on the lower isoprene emission from children. The concepts proposed here could be tested more thoroughly if more films are sampled. In particular, a larger suite of films with rating “FSK 16” and “FSK 18” would be interesting as they represent the extreme categories.

## Supporting information

S1 Table Summary of the attendees statistic. The numbers show the average amount of viewers attending the showroom. **Detailed description of the box model**.
(DOCX)

S2 Table Mean values of the extracted features. (TXT)

S3 Table Standard deviation of the extracted features. (TXT)

S4 Table Summary of the area under ROC curve calculated for all VOCs. (TXT)

S5 Table Summary of the standard deviation of the area under curve for all measured VOCs. (TXT)

S6 Table Summary of the p-value derived from the permutation test for all measured VOCs. (TXT)

S7 Table Ticket sales data for winter 2013/2014. All dates in the ticket sale data and in the compounds tables are in local time (UTC+1). The units in the compound S9 and S10 Tables are in ppb besides for CO_2_ which is in ppm.
(TXT)

S8 Table Ticket sales data for winter 2015/2016. All dates in the ticket sale data and in the compounds tables are in local time (UTC+1). The units in the compound S9 and S10 Tables are in ppb besides for CO_2_ which is in ppm.
(TXT)

S9 Table Measured compounds for winter 2013/2014. All dates in the ticket sale data and in the compounds tables are in local time (UTC+1). The units in the compound S9 and S10 Tables are in ppb besides for CO_2_ which is in ppm.
(TXT)

S10 Table Measured compounds for winter 2015/2016. All dates in the ticket sale data and in the compounds tables are in local time (UTC+1). The units in the compound S9 and S10 Tables are in ppb besides for CO_2_ which is in ppm.
(TXT)
