# Supplementary material for: Proof of concept study: Testing human volatile organic compounds as tools for age classification of films
Source: PLoS One. 2018 Oct 11;13(10):e0203044. doi: 10.1371/journal.pone.0203044 (PMC6181293; doi:10.1371/journal.pone.0203044)
Supplement: S1 Table — The numbers show the average amount of viewers attending the showroom. Detailed description of the box model. (DOCX) [file pone.0203044.s001.docx]

Supplement: Can the age classification of films be made based on audience breath-chemical emissions?

**S1 Table**: **Summary of the attendees statistic.** The numbers show the average amount of viewers attending the showroom.

| FSK 0 | FSK 6 | FSK 12 | FSK 16 |
| --- | --- | --- | --- |
| Help, I've shrunk my teacher 55 | Buddy 104 | The Starving Games 52 | Counselor 90 |
| I'm off then 122 | Dinosaurs 3D 36 | Hunger Games: Catching Fire 118 | Machete Kills 44 |
|  | Walter Mitty 138 | Star Wars: The Force Awakens 97 | Paranormal Activity: Ghost Dimension 130 |

**Detailed description of the box model**

The modelled mixing ratios for the compounds were calculated by applying a mass-balance-approach. In order to use this model several assumptions must be made including that there is no pathway for mass loss except air exchange and that the emission rate *p* is small compared to the air exchange rate. It was observed that the air was less effectively mixed in the lower part of the cinema. Thus a mixing factor *q* must be introduced accounting for the incomplete mixing of the air.^1^ The volume of the screening room was 1300 m^3^ and the air supply was 6500 m^3^/h with identical flows in and out of the showroom provided by the software control of the ventilation system (Instatec Klima-Energietechnik GmbH). Equation 1 shows the ordinary differential equation (ODE) which must be solved optimizing the parameters *q* the mixing factor and *p* the emission rate.

*dm/dt = c_in_ ·q · r · + p - c_out_ · q · r* (1)

In equation 1, *m* is the mass of the molecules at time *t* in the screening room air. The outside air is supplied with a ventilation rate *r* and a mixing ratio *c_in_*. The mixing ratio c*_in_* was interpolated from the two surrounding background night time measurements in the absence of people for each VOC. To account for the imperfect air mixing The ventilation rate *r* is multiplied with the mixing parameter *q* and the product (*q* · *r*) provides a smaller effective room ventilation rate. Consequently, the lower the mixing factor q the worse the mixing of air in the room. The emission rate of a given gas from the audience is given by *p*.

The estimation of the emission rate *p* and the mixing factor *q* involves the solving of the ordinary differential equation shown as equation 1. The optimization was performed using a non-linear least squares method. The estimated constant emission rate *p* of the VOC and the mixing factor *q* were used to calculate the mixing ratio of the VOC, which could be seen as the red curve on the left side in Figure 1 in the manuscript.
